# Influence of Different Diets on Growth and Development of Eastern Honey Bee (*Apis cerana*)

**DOI:** 10.3390/insects16040383

**Published:** 2025-04-03

**Authors:** Ruonan Liang, Cheng Liang, Yi Zhang, Jiaxing Huang, Guiling Ding

**Affiliations:** 1 State Key Laboratory of Resource Insects, Institute of Apicultural Research, Chinese Academy of Agricultural Sciences, Beijing 100193, China; 2 Sericulture and Apiculture Research Institute, Yunnan Academy of Agricultural Sciences, Mengzi 661101, China; 3 School of Chinese Medicinal Resource, Guangdong Pharmaceutical University, Yunfu 527527, China

**Keywords:** pollen, pollen substitute, diet consumption, body weight, survival, hypopharyngeal gland, midgut proteolytic enzyme activity

## Abstract

In recent years, honey bees have been stressed by multiple factors, with malnutrition posing a significant threat to the healthy development of honey bee colonies. To keep a colony healthy and productive, beekeepers supply their colonies with supplementary pollen or commercial pollen substitutes during periods of pollen dearth or insufficient pollen quantity or quality. In this study, we evaluated the effects of four natural pollen types (oilseed rape pollen, camellia pollen, lotus pollen and buckwheat pollen) and two commercial pollen substitutes (Diet 1 and Diet 2) against a control group (sucrose solution) on *Apis cerana* through cage experiments. The food consumption, live body weight, longevity, hypopharyngeal gland development and midgut proteolytic enzyme activity of caged workers were measured. The food consumption rates of oilseed rape pollen and buckwheat pollen were greater than the other diets. Oilseed rape pollen and camellia pollen were recognized as excellent-quality diets for hypopharyngeal gland development and midgut proteolytic enzyme activity. Over the entire experimental period, the caged workers fed with lotus pollen had a similar diet consumption and body weight to those fed with pollen substitutes, and these bees had a significantly higher survival rate than those fed with other diets. The results indicated that the commercial pollen substitutes appeared to be less beneficial to caged *A. cerana* workers than the natural pollen resources.

## Simple Summary

Nowadays, honey bees are confronted by many stressors, such as agricultural intensification, habitat loss, pesticide use, climate change and diseases. These factors make malnutrition a significant threat to the healthy development of honey bee colonies. Beekeepers need to provide pollen or a pollen substitute during a pollen dearth period to support the development of colonies. Our study compared the effects of four natural pollen resources and two pollen substitutes against a control group on *Apis cerana* workers through cage experiments. The diet consumption, diet preference, body weight, survival, hypopharyngeal gland development, and midgut proteolytic enzyme activity of caged workers were assessed. Our study revealed that *A. cerana* workers showed a preference for oilseed rape pollen and buckwheat pollen. The workers fed with oilseed rape pollen exhibited the highest body weight and the optimal development of hypopharyngeal gland. However, the workers supplied with lotus pollen had a significantly higher survival rate than those fed with other diets. In addition, our study demonstrated that the pollen substitutes commonly used in China were not as beneficial as most natural pollen resources to *A. cerana* caged workers.

## 1. Introduction

Honey bees are important pollinators of crops and wild plants [1]. They play an important role in maintaining ecological balance and species diversity [2]. Honey bees collect pollen and nectar from flowering plants to absorb nutrients. Nectar is the main source of carbohydrate, while pollen acts as the primary source of protein, and both of them are necessary for the survival and development of honey bees [3]. Honey bees need an adequate pollen quantity and quality to remain healthy and productive [4]. But nowadays, honey bees are confronted by many stressors which result in pollen deficiencies. These stressors, such as changes in landscape use [5], agricultural intensification [6], seasonal pollen dearth and adverse weather conditions [7,8], may prevent honey bees from foraging a sufficient diversity, quantity or quality of pollen. To keep a colony healthy and productive, beekeepers supply their colonies with supplementary pollen or commercial pollen substitutes during periods of pollen dearths or insufficient pollen quantity or quality.

The Western honey bee (*Apis mellifera*) is native to Europe and Africa, while the Eastern honey bee (*Apis cerana*) is native to Asia [9]. Both *A. mellifera* and *A. cerana* are important pollinators in many natural ecosystems and agro-ecosystems [1,10]. *A. mellifera* is the globally predominant managed bee species and has a strong foraging orientation and exhibits high performance regarding bee products [11]. In China, there are approximately 9 million managed colonies (about 6.8 million of *A. mellifera* colonies and more than 2 million of *A. cerana* colonies) and more than three hundred thousand beekeepers [12]. *A. mellifera* is distributed throughout all the country and dominates the production of bee products, such as honey, royal jelly, pollen and propolis. *A. cerana* is an important indigenous species and is raised extensively in the mountainous regions, especially in southern and eastern China [13]. It is both wild and partly managed by beekeepers. The colony size of *A. cerana* is smaller than that of *A. mellifera*, but *A. cerana* outperforms *A. mellifera* with respect to collecting scattered nectar resources. Colony reproduction and honey are the most important sources of income for *A. cerana* beekeepers [14].

There are two nectar flow periods in southern China (from March to June and from late August to January of the following year). In other regions of China, the nectar flow can last from April to September. In southern regions, honey bees face pollen and nectar scarcity primarily in March and between July and August [15]. In some regions of Yunnan Province, the rainy season can last from June to October. The prolonged rainy season hinders bees’ foraging activities, which can result in food scarcity. During this period, beekeepers supply their colonies with feed supplements to stimulate queens to lay eggs to avoid the subsequent decline or absconding of colonies. In eastern, central and northwestern China, the scarcity periods can occur during autumn, winter and spring. Feeding with pollen or a pollen substitute usually takes place during periods of pollen scarcity [15]. At present, supplying colonies with pollen or a pollen substitute is a common practice in China to promote a faster buildup in early spring, to extend the life of pollinating colonies in greenhouses, to ensure successful overwintering in early winter, to increase royal jelly yield during the production season, and to alleviate colony decline in hot summers [16].

China is the largest pollen producer in the world [17] and the annual pollen production is estimated to be approximately 5000 metric tons [18]. In China, most of the territory has a temperate climate, with sub-tropical and tropical areas in the south and a cold temperate region in the north. This varied ecological environment maintains floral resources diversity for honeybees. The most common types of Chinese monofloral bee pollen collected by *A. mellifera* include oilseed rape (*Brassica napus* L.) pollen, buckwheat pollen (*Fagopyrum esculentum* Moench), sunflower pollen (*Helianthus annuus* L.), lotus (*Nelumbo nucifera* Gaertn) pollen, wuweizi (*Schisandra chinensis*) pollen, camellia (*Camellia sinensis* L.) pollen, and so on [17,19]. Given the expensive cost and disease transmission risks associated with pollen [20], it is important to develop effective pollen substitutes that can be provided to colonies to mitigate the lack of natural pollen resources.

The earliest published study on pollen substitutes was conducted in the early 1900s in the U.S. and Canada [21]. In China, beekeepers usually provided stored pollen combs during periods of pollen scarcity before the 1980s. Later, soy flour was used instead of pollen combs. But soon, beekeepers noticed that these simple artificial feeds reduced bee product production and bee immunity due to insufficient nutritional supply. At present, most beekeepers use pollen pellets collected by *A. mellifera* or artificial pollen substitutes supplied on the market to supplement protein diets [22]. Although research into bee feeds in China is in its initial stage, enterprises that sell protein diets that claim to be formulated for *A. mellifera* or *A. cerana* have been growing fast in recent years. There are mainly two types of commercial protein diets provided on the market. One is the powder type, where beekeepers add sugar, honey and water to make a paste feed for bees to eat. The other is the paste type made by a manufacturer and sold to a beekeeper for direct use. Although these diets have been widely used, no data are available concerning their effects on honey bees’ health. A survey on colony winter losses in China revealed that the mortality of bees in Henan Province has continued to remain at a high level compared to other provinces [12,23]. It was suggested that the high mortality in Henan Province may be related to the extensive use of pollen substitutes [23]. Currently, more and more beekeepers are expressing concerns about the effectiveness of these diets available on the market.

As the primary source of dietary proteins and lipids for honey bees, pollen is important for their gland and organ development [24], longevity [10,25,26], and immunocompetence [27]. Previous studies have compared the effects of different types of pollen or pollen substitute diets at the levels of immunocompetence [28,29], survival [30,31], the physiology of workers [3,32], ovarian activation [33,34], the development of larvae [35,36], consumption rates by colonies, and their effects on brood and adult populations [37,38]. The pollen of different host–plant species varies greatly in nutritional content [39,40]. Such variability in the nutritional composition and quantity of pollen contributes to its different influences on honey bees at the individual or colony level [3,27].

An effective protein diet should be attractive to honey bees and benefit their overall health. Hence, we need to use combined indicators to evaluate the overall value of a diet. Diet consumption is a key indicator of diet quality, attractiveness, and effectiveness [41]. Dietary preference could provide insight into the palatability and immediate acceptability of a diet [42]. The midgut of bees is the primary site where they digest and absorb protein [43]. The protein is hydrolyzed by proteolytic enzymes in the midgut, and the nutrients are converted into royal jelly by the hypopharyngeal glands. The hypopharyngeal glands are the main glands of young bees that convert the nutrients derived from pollen into royal jelly, which is an essential food for the development of larvae and queens [44]. Hypopharyngeal gland development and protein production and midgut proteolytic enzyme activity are affected by protein diets [37,45]. In addition, longevity and body weight are also important indicators used to assess diet quality [46,47,48,49].

In this study, we conducted a controlled laboratory experiment to investigate the effects of different diets on *A. cerana* workers. We compared the effects of four common species of monofloral bee pollen (oilseed rape pollen, camellia pollen, lotus pollen, and buckwheat pollen) and two widely used commercial pollen substitutes (Diet 1 and Diet 2) against a control group on caged honey bees. We assessed the diet consumption, diet preference, body weight, survival, acinus surface area and protein content of the hypopharyngeal gland and the total midgut proteolytic enzyme activity. This study aims to explore the influence of pollen pellets and pollen substitutes on *A. cerana* workers. We hypothesized that different diets would display different effects and natural pollen would be more beneficial to the workers than pollen substitutes. The results can help us better understand the fundamental aspects of the physiological responses of *A. cerana* to different diets, which is helpful for the development of more effective diets.

## 2. Materials and Methods

### 2.1. Honey Bee Rearing and Feeding

This study was conducted in Caoba during November 2023 and January 2024 at the Sericulture and Apiculture Research Institute, the Yunnan Academy of Agricultural Sciences. This area is characterized by a mountainous and plateau terrain, featuring a subtropical plateau monsoon climate. The average daytime temperature in winter is above 15 °C. During the period of this study, the main flowering plants included eucalyptus, loquat, and some other wild plants. In this area, the brood production of colonies could last all year round.

Frames of sealed brood collected from four queenright *A. cerana* colonies were placed into an incubator at 34 °C and 75% relative humidity. The workers that emerged within 24 h were collected and mixed before placing them into cages, with 30 workers per cage.

**Figure 1:**
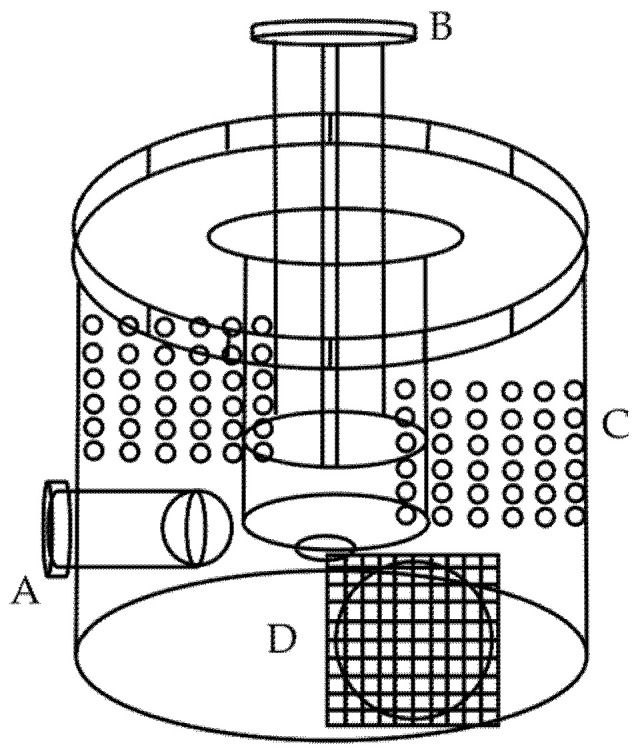
A model of the experimental cage used in the study. (A) The Eppendorf tube with the protein diet; (B) the syringe containing sucrose solution; (C) ventilation holes; (D) the sampling hole.

The experimental cages were modified according to Atreya et al. [50]. They were made from 420 mL plastic cups. Holes were drilled on the plastic cup using a borer. One hole was in the top of the cage to accommodate a 5 mL syringe containing sucrose solution. The others were on the side of the cage to accommodate 1.5 mL Eppendorf tubes with their tips cut for the workers to acquire the protein diets. A hole with a 2 cm diameter was drilled, sealed with a screen cloth, and used for sampling. We also drilled 36 holes 2 mm in diameter on the sides of the cage for ventilation (Figure 1).

The caged workers were maintained in an incubator at 30 °C and 50% relative humidity. There were three cages each for the pollen-fed treatment, the pollen substitute-fed treatment and the control. Workers of the treatment groups were fed with 50% (*w*/*w*) sucrose solution and supplied with one of the six types of protein pastes, i.e., oilseed rape pollen, camellia pollen, lotus pollen, buckwheat pollen, pollen substitute #1 (Diet 1) and pollen substitute #2 (Diet 2). The control group was supplied with only 50% sucrose solution ad libitum to provide carbohydrates.

The four types of corbicular pollen were collected in 2023 from *A. mellifera* hives outfitted with pollen traps. These dried pollen pellets were stored at −20 °C until used. In addition, pesticide residues including acrinathrin, carbofuran, cyfluthrin, deltamethrin, dichlorvos, fenitrothion, fenopropathrin, imidacloprid, and acetamiprid were checked and all of them were not detected within these pollen pellets. Detailed information about the bee pollen used in this study is shown in Table 1.

**Figure 2:**
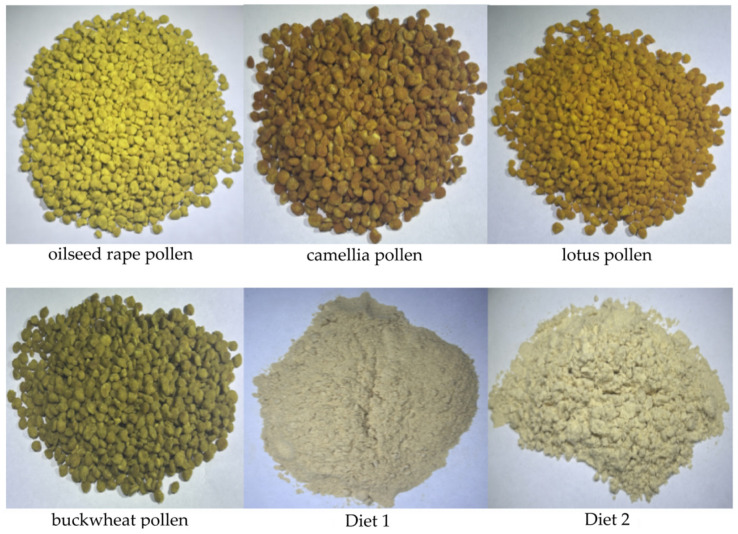
The pollen pellets and pollen substitutes used in this study.

For the natural pollen pellets (oilseed rape-dominant, camellia-dominant, lotus-dominant and buckwheat-dominant blends), we firstly hand-sorted the pollen loads based on their color and appearance. Then, we confirmed the purity of the pollen by checking its morphological uniformity under a microscope. Using this manual sorting, the purity of the pollen pellets was 95% or above (Figure 2).

Diet 1 was purchased from Jingjiang Feng Yun Bee Feed Co., Ltd. (Jingjiang, China), and Diet 2 was obtained from Sichuan Wei Feng Biotechnology Co., Ltd. (Deyang, China). These artificial diets are said to be formulated for *A. cerana* and they are widely employed by beekeepers. Both of them are the powder type (Figure 2). Detailed information about the pollen substitutes is shown in Table 2.

To prepare the feed paste, we mixed pollen pellets with 50% sucrose solution at a ratio of 5:2.8 (*w*/*v*). Each diet was made by thoroughly mixing the pollen with sucrose solution until a kneadable and stable texture was obtained. Each time, approximately 1.5 mL of 50% sucrose solution and 2 g of protein diets were provided to the workers in each cage. These amounts of the diets ensured that there was some left over when the diets were refreshed. For each test, three additional bee-free cages were implemented with each protein diet and 50% sucrose solution to control for evaporation and adjust the data. Every day, we replaced the sucrose solution and protein diets at 9:00 a.m. to make sure that fresh diets were supplied to the honey bees.

### 2.2. Food Consumption, Body Weight and Survival Analysis

We weighed the feeders loaded with the protein diet before inserting them into the cages and after removing them from the cages. With the evaporation loss subtracted, the daily protein diet consumption was calculated by subtracting the pre-feeding weight from the post-feeding weight. Each bee’s daily consumption of the protein diets was calculated by dividing the daily diet consumption by the number of living bees.

To analyze the fresh body weight of the workers, we randomly selected 10 surviving workers from each cage on days 1, 5, 10, 15, and 20 and recorded the body weight of individual honey bees.

Throughout the experiment, the surviving workers were counted and provided with fresh food on a daily basis and the dead bees were removed from the cages. The experiment lasted until the mortality of the workers of all the groups reached 50% or above.

### 2.3. Diet Preferences

We conducted a choice test to determine the relative preference of workers for the protein diets. Four cages of newly emerged workers collected from frames of one colony were prepared and monitored. In each cage, all the six different protein diets were supplied simultaneously. Six Eppendorf tubes were placed at a distance of 2 cm, with a single diet placed into a single 1.5 mL tube. Each protein diet’s consumption was recorded daily as stated above. We also recorded the number of workers consuming each diet at 11:00 and 15:00 under a red light for 5 continuous days. Each time, we observed for 5 min and counted the number of workers that contacted a particular protein diet with their proboscis or mandibles.

### 2.4. Hypopharyngeal Gland Measurements

On days 5, 10 and 15, we randomly selected 10 workers from each cage. The heads of workers were dissected and a pair of hypopharyngeal glands were removed. The glands were placed on a slide with a drop of 0.25 M NaCl solution (Shanghai Yuanye Biotechnology Co., Ltd., Shanghai, China). For each worker, the maximum length and width of ten randomly selected acini were measured using a Leica DM 3000 microscope (Leica, Wetzlar, Germany) equipped with LAS V4.13 software. The measurement was taken at a 400× magnification, using a 200 μm scale bar. For the measurement, we only selected the acini with clearly defined borders. The acinus size was averaged within each individual. The area of each acinus was calculated using the following formula [51]:

$$\mathrm{Acinus}\;\mathrm{surface}\;\mathrm{area}=\frac{a\times b}{2}\times \pi ,$$

where a is the maximum length, b is the maximum width of the acinus, and π = 3.14.

To measure the total protein content, the hypopharyngeal glands of ten workers from each cage were placed together. They were homogenized in 1.5 mL Eppendorf centrifuge tubes containing 100 μL lysis buffer (50 mM Tris pH 7.4, 150 mM NaCl, 1% NP-40) including a protease and phosphatase inhibitor cocktail (Beyotime, Shanghai, China). Then, they were placed in an ice bath for 30 min. After centrifugation at 12,000× *g* at 4 °C for 10 min, the supernatant was collected and transferred to a new centrifuge tube. The protein concentration was determined using an Enhanced BCA Protein Assay Kit (Beyotime, Shanghai, China) on a microplate reader (Synergy Neo2, BioTek, Winooski, VT, USA). Firstly, we prepared the standard solution of a known concentration using the reagents provided in the kit. The absorbance was measured and a standard curve was made. Then, the supernatant of the samples was added to working solution and the absorbance was assessed at 562 nm. Finally, the protein concentration of the samples was calculated based on the standard curve and the volume of the samples used.

### 2.5. Assessment of Total Midgut Proteolytic Enzyme Activity

The workers collected on days 5, 10 and 15 were used both for the assessment of the development of the hypopharyngeal gland and the total midgut proteolytic enzyme activity. The enzyme activity was determined using a commercial Pepsin Assay Kit (NJJC, Nanjing, China). Firstly, the workers were cold-anesthetized and sterilized using 75% ethanol. Then, the midguts of 10 randomly selected workers per cage were isolated by dissecting. These midguts were weighed and homogenized at 4 °C in the buffer solution (provided in the kit) at a ratio of 1:9 (*w*/*v*). The mixtures were centrifuged at 2500 rpm for 10 min. The supernatant was used to determine protease activity following the instructions of the kit on a spectrophotometer (752 UV VIS, Shanghai Jinghua, Shanghai, China). The absorbance was measured at a wavelength of 660 nm. Each sample was assessed three times and the average value was taken as the final result.

### 2.6. Statistical Analysis

All data were expressed as mean ± standard error when applicable and analyzed using IBM SPSS statistics 29. The data were tested for normality using Shapiro–Wilk’s test and Levene’s test for the homogeneity of variances. The differences in diet consumption, acinus surface area and protein content of the hypopharyngeal glands and midgut proteolytic enzyme activity were compared using the analysis of variance (ANOVA). The feeding preference (diet consumption) and body weight were tested using generalized linear mixed models (GLMMs), and the feeding preference (bee number) was analyzed using a generalized linear model (GLM). Post hoc multiple comparisons were made using Tukey’s multiple comparison test and differences were considered significant at *p* values < 0.05. The percent survival of caged workers was analyzed using Kaplan–Meier survival curves and statistical differences were evaluated using a log-rank test.

## 3. Results

### 3.1. Diet Consumption

**Figure 3:**
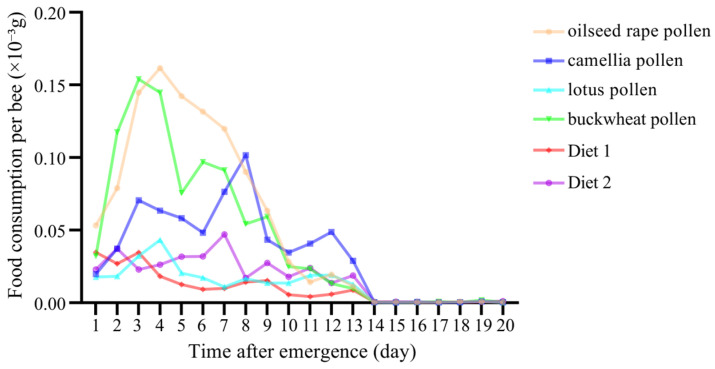
Daily consumption of diets by caged workers over a period of 20 days.

The diets were mainly consumed by the caged workers at the age of 1–13 days. The highest consumption rate was on day 3 for buckwheat pollen and on day 4 for oilseed rape pollen and lotus pollen. For Diet 2 and camellia pollen, the highest consumption rate was, respectively, on day 7 and day 8 (Figure 3). The food consumption rates of oilseed rape pollen and buckwheat pollen were greater than the other groups, while a lower consumption rate was recorded in caged workers provided with lotus pollen, Diet 1 and Diet 2 (Figure 3). In addition, there were no significant differences in food consumption among all the groups on day 1, from day 8 to day 10, and after the 12th day. No significant differences were detected in food consumption among workers fed with lotus pollen, Diet 1 and Diet 2 or between workers fed with oilseed rape pollen and buckwheat pollen (*p* > 0.05).

### 3.2. Diet Preference

The total consumption was significantly different among the six protein diets (*F*_5, 15_ = 7.460, *p* = 0.001). The workers consumed significantly more buckwheat pollen (0.357 ± 0.092 g) than lotus pollen (0.117 ± 0.008 g) and Diet 1 (0.135 ± 0.002 g). Oilseed rape pollen, camellia pollen and Diet 2 were consumed similarly to buckwheat pollen (Table 3). The number of workers consuming buckwheat pollen (13.0 ± 3.4) was significantly greater than those consuming other protein diets (*F*_5, 15_ = 10.082, *p* < 0.001) (Table 3).

### 3.3. Body Weight

**Figure 4:**
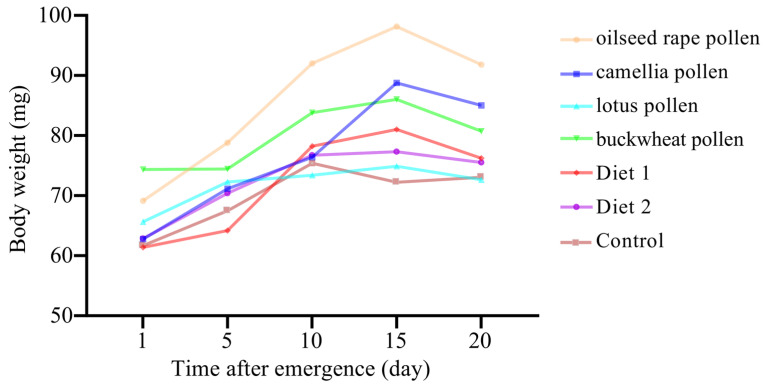
The body weight of caged workers fed with different diets.

There were significant differences in the body weight among diet treatments at different times (*p* < 0.05). When the experiment started, the mean live body weights ranged from 0.060 ± 0.007 g (the group of workers fed with Diet 1) to 0.075 ± 0.011 g (the group of workers fed with buckwheat pollen). There were significant differences among diet treatments at different ages (*F*_4, 24_ = 39.718, *p* < 0.001). At the beginning of the experiment, workers of the group fed with buckwheat pollen were significantly heavier than those of other groups (*p* < 0.05). Then, the body weight of workers fed with oilseed rape pollen increased faster. From the 5th day, workers fed with oilseed rape pollen were significantly heavier than those fed with Diet 1, Diet 2 and the sucrose solution (*p* < 0.05). No significant difference was detected in body weight among workers fed with lotus pollen, Diet 1, Diet 2 and the control (*p* > 0.05). The body weight of caged bees fed with protein diets increased until the 15th day (ranged from 0.072 ± 0.015 g to 0.098 ± 0.007 g). For the control group, the highest body weight was recorded on the 10th day (Figure 4).

### 3.4. Survival Probability

**Figure 5:**
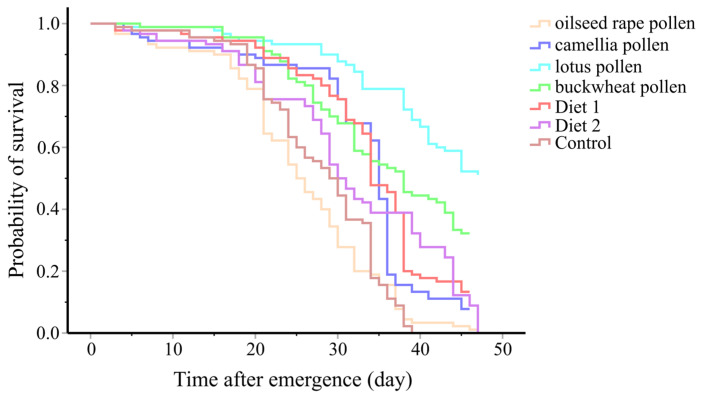
The survival probability of workers supplied with different diets.

The Kaplan–Meier survival curves revealed significant effects of the diets on the mortality of caged workers (Figure 5). The workers supplied with lotus pollen (LT_50_ = 47.0 days) had a significantly higher survival rate than the workers fed with other diets (*p* < 0.001). The workers supplied with oilseed rape pollen (LT_50_ = 24.0 days) were estimated to have the lowest survival rate, which was not significant compared to the workers fed with sucrose solution only (LT_50_ = 28.0 days) (χ^2^ = 0.870, *p* = 0.351). There was no significant difference in the mortality between workers that consumed Diet 1 (LT_50_ = 33.0 days) and Diet 2 (LT_50_ = 29.0 days) (χ^2^ = 0.756, *p* = 0.385), camellia pollen (LT_50_ = 34.0 days) and Diet 1 (χ^2^ = 2.037, *p* = 0.153), and camellia pollen (LT_50_ = 34.0 days) and Diet 2 (χ^2^ = 0.004, *p* = 0.948). After 47 days, a mortality rate of 100% was observed in the workers fed with oilseed rape pollen, Diet 2 and sucrose solution, while the mortality rate was 92.22%, 50%, 67.78% and 86.67% for camellia pollen, lotus pollen, buckwheat pollen and Diet 1, respectively (Figure 5).

### 3.5. Hypopharyngeal Gland Development

**Figure 6:**
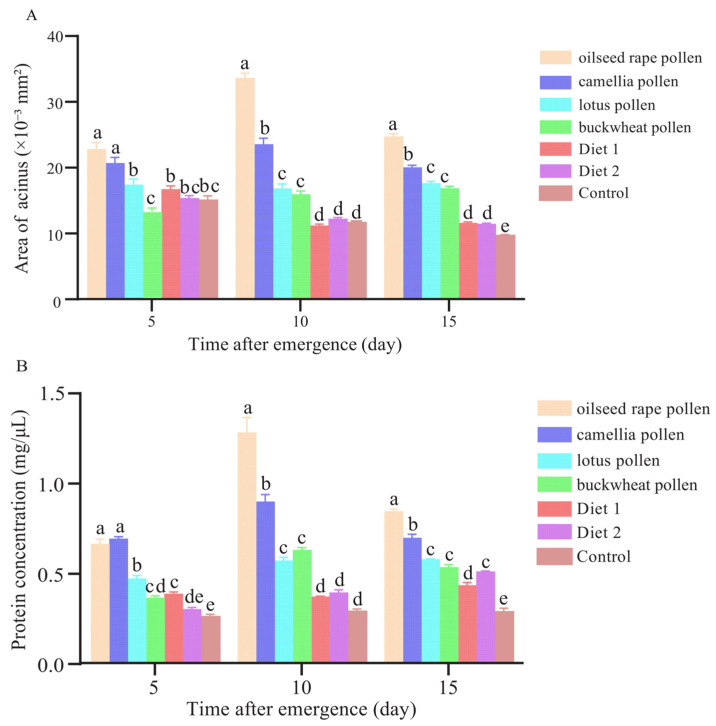
The development of the hypopharyngeal gland in adult workers fed with different diets. (**A**) A comparison of the surface area of acinus at different time points; (**B**) a comparison of the protein content at different time points. Different letters indicate significant differences among the treatments (*p* < 0.05).

The average area of the acinus of the hypopharyngeal gland was dependent upon the type of diets (*F* = 300.071, *p* < 0.001) and the age of workers (*F* = 21.743, *p* < 0.001). There were significant interactions between diet and age (*F* = 30.844, *p* < 0.001). On day 5, the acini were significantly smaller in workers fed with buckwheat pollen than those fed with oilseed rape pollen, camellia pollen, lotus pollen and Diet 1 (*p* < 0.001). The workers fed with oilseed rape pollen had a similar size of acini to those fed with camellia pollen (*p* > 0.05). Both of them were significantly larger than the workers supplied with other diets (Figure 6A). On day 10 and day 15, the acini were similar in workers fed with lotus pollen and buckwheat pollen (*p* > 0.05). On day 15, the control had significantly smaller acini than the treatments (*p* < 0.05). On days 5, 10 and 15, the acini size of the workers fed with Diet 1 did not differ from those fed with Diet 2 (*p* > 0.05). The acinus area of workers fed with oilseed rape pollen and camellia pollen was significantly larger on day 10 than those on day 5 or day 15 (*p* < 0.001). For the workers fed with lotus pollen, the acini surface area was not significantly different at different times (*p* > 0.05) (Figure 6A).

The protein content of the hypopharyngeal glands was influenced by the type of diets (*F* = 932.041, *p* < 0.001) and the age of workers (*F* = 365.248, *p* < 0.001). There were significant interactions between diet and age (*F* = 88.686, *p* < 0.001). The workers fed with oilseed rape pollen showed the highest protein concentration, while the control showed the lowest (Figure 6B). On days 5, 10 and 15, workers fed with oilseed rape pollen and camellia pollen showed a significantly higher protein concentration than the others (*p* < 0.001). On day 10, the protein concentration of workers supplied with oilseed rape pollen, camellia pollen and buckwheat pollen was significantly higher than those on day 5 and day 15 (*p* < 0.001). On day 5 and day 10, no significant differences were observed in the protein concentration among the workers supplied with Diet 1 and Diet 2 and the control (*p* > 0.05) (Figure 6B).

### 3.6. Total Midgut Proteolytic Enzyme Activity

**Figure 7:**
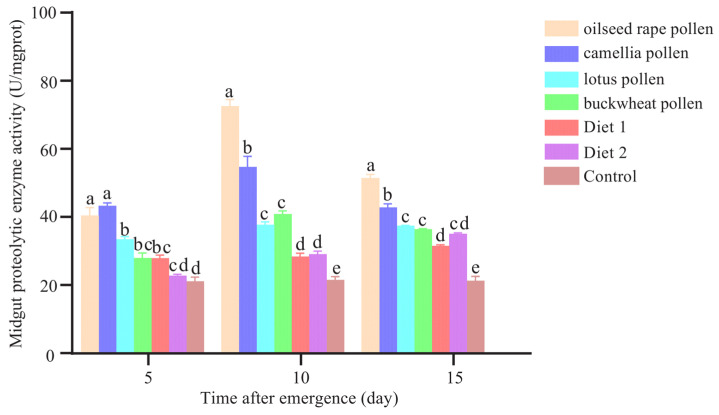
The total midgut proteolytic enzyme activity of workers fed with different diets. Different letters indicate significant differences among the treatments (*p* < 0.05).

The midgut proteolytic enzyme activity was influenced by the type of diets (*F* = 248.059, *p* < 0.001) and the age of workers (*F* = 105.046, *p* < 0.001). There were significant interactions between diet and age (*F* = 24.967, *p* < 0.001). The workers fed with oilseed rape pollen showed the highest midgut proteolytic enzyme activity, while the control group had the lowest (Figure 7). On day 5, workers fed with oilseed rape pollen and camellia pollen showed significantly higher midgut proteolytic enzyme activity than the others (*p* < 0.001). On day 10, the midgut proteolytic enzyme activity of workers fed with oilseed rape pollen was significantly higher than that on day 5 and day 15 (*p* < 0.001). There were no significant differences observed between workers fed with lotus pollen and those fed with buckwheat pollen or between Diet 1 and Diet 2 (*p* > 0.05) (Figure 7).

## 4. Discussion

The results obtained in this study revealed different effects of the protein diets that we assessed on *A. cerana* caged workers. Workers supplied with oilseed rape pollen and buckwheat pollen showed greater food consumption rates than those fed with the other diets. Workers fed with lotus pollen had a similar diet consumption and body weight to those fed with Diet 1 and Diet 2 over the entire experimental period. However, workers fed with lotus pollen showed the best survival, while those fed with oilseed rape pollen had the lowest survival rate. Workers fed with oilseed rape pollen and camellia pollen had a greater acinus size and protein content in the hypopharyngeal glands and midgut proteolytic enzyme activity. Our study highlighted the importance of protein diets for the survival, body weight, hypopharyngeal gland development and midgut proteolytic enzyme activity of *A. cerana* bees. Our study also demonstrated that natural pollen resources are more beneficial to *A. cerana* workers than the pollen substitutes mostly used in Chinese apiculture.

As previously reported, this study demonstrated that the type of diets and the age of workers significantly influence the development of the hypopharyngeal glands [52] and midgut proteolytic enzyme activity [45]. For the workers fed with natural pollen resources, the acinus size and protein content of hypopharyngeal glands and the midgut proteolytic enzyme activity peaked on day 10, while a slightly different temporal pattern was found for workers fed with pollen substitutes of Diet 1 and Diet 2. Peng et al. [53] demonstrated that the workers fed with oilseed rape pollen exhibited superior hypopharyngeal gland development. Consistent with this previous report, our study showed that the acinus size and protein content of hypopharyngeal glands and the midgut proteolytic enzyme activity of the workers fed with oilseed rape pollen were significantly higher than those fed with other diets. This indicated that nurse bees supplied with oilseed rape pollen could be better producers of brood food as a result of the better development of their hypopharyngeal glands. Compared with other diets, oilseed rape pollen is more suitable for the growth and development of bees.

The control group of workers fed with sugar syrup alone had a lower protein concentration and smaller acinus in the hypopharyngeal glands and significantly lower midgut proteolytic enzyme activity compared to those fed the other diets treatments, which is similar to the results reported by previous studies [54,55,56]. This implies that protein diets are necessary for the development of hypopharyngeal glands [43]. Previous studies have reported that proteolytic enzyme activity is influenced by protein levels of the food [37,57], and ratios of protein to lipids in the diets might influence hypopharyngeal gland growth [58]. Although buckwheat pollen had a lower protein content than lotus pollen, the protein content in the hypopharyngeal glands and the midgut proteolytic enzyme activity of workers fed with buckwheat pollen was higher than those fed with lotus pollen. This may be attributed to the diverse amino acid composition of buckwheat pollen, particularly exogenous amino acids, which enhance its nutritional value [59]. Further studies analyzing the nutritional ingredients of diets may provide more insight into the differences in hypopharyngeal gland development and midgut proteolytic enzyme activity among different diet groups observed in this study.

In our study, significant differences in feeding preference were observed among the diets. According to Manaswi et al. [50], caged *A. mellifera* workers have a strong preference for wildflower pollen over commercially available pollen substitutes. However, our study indicated that the natural lotus pollen was just as unattractive to *A. cerana* workers as pollen substitutes. The differences in pollen and bee species and in the composition of pollen and pollen substitutes possibly lead to the difference in feeding preference. In a field study with *A. mellifera*, it was indicated that the first criteria for the honey bee foraging preference of pollen should be the nutritional contents of protein and the resource availability of less nutritious floral sources [60]. In our study, the protein content was significantly different among the diets. The highest protein content was found in Diet 2 and the lowest protein content was found in buckwheat pollen. However, our result revealed that buckwheat pollen was the most preferred. Therefore, no positive correlation between worker feeding preference and the protein content of diets was found in our study. Previous studies have suggested that several factors including physical and chemical cues could influence diet attractiveness. A strong correlation between pollen phagostimulants and foraging preference was found in Boch [61]. In the study of Bertazzini et al. [62], it was revealed that honey bees preferred proline-containing artificial nectars to alanine and serine and alanine to serine. Recently, Kim et al. [63] reported that histidine exhibited a highly positive correlation with the preference for and digestibility of a pollen substitute diet. Hence, further studies are needed to elucidate what factors are at play in the attractiveness difference in the protein diets in this study.

Significant differences were observed in the consumption of various diets, while the consumption rate patterns were similar for all the tested diet groups. Generally, the workers consumed more natural pollen than pollen substitutes, which is consistent with previous studies [54,57,64]. The protein diets were mainly consumed by workers aged 1 to 9 days and the consumption nearly stopped by day 14. These results are concordant with previous studies that revealed that bees consume a pollen diet mainly at the age of 1–9 days [65,66] and the consumption of proteinaceous diets decreases sharply and stops at a low level by day 15 [32,52]. Our study indicated that the consumption of oilseed rape pollen and buckwheat pollen was much greater than that of lotus pollen, which was consumed at a similarly low level to the pollen substitutes. In a field study with *A. mellifera*, the colonies consumed significantly greater amounts of oilseed rape pollen and camellia pollen than lotus pollen [67], which is somewhat consistent with our laboratory findings.

As an indicator of honey bee health, the body weight of bees has been measured in various studies. In this study, we noticed that the workers fed with oilseed rape pollen were heavier than those fed with other diets. In addition, the body weight of workers fed with oilseed rape pollen increased faster than those fed with other diets from day 5. This could be due to that the food consumption rates of oilseed rape pollen were greater than the other groups. As it was stated previously, there was a significant positive correlation between the sum of paste consumption and individual body weight [47].

Numerous studies have shown that while high-protein pollen aids bee development, excessive or poor-quality protein sources may shorten bee lifespan [68,69]. Among all the diets, oilseed rape pollen and Diet 2 have a higher protein content. However, workers fed with these diets all died on the 47th day. This may be due to nutritional excess leading to the accumulation of undigested substances in their bodies, which in turn limits their physiological functions and accelerates mortality [37]. The protein content of lotus pollen was higher than that of buckwheat pollen but lower than that of oilseed rape pollen. The longevity of workers was the longest in those fed lotus pollen, followed by buckwheat pollen, and the shortest was in the oilseed rape pollen group. This indicated that bee lifespan is not solely related to the protein content in the feed. It might also be influenced by the composition and proportion of nutritional components [32,70], particularly the requirements for specific essential amino acids, dietary protein concentration, and the protein-to-lipid (P:L) ratio [71].

The nutritional content varies greatly among different pollen types [17]. The composition of bee pollen may also be affected by differences in gathering area or time [72]. Different processes or storage treatments in commercial production could also introduce variations in the composition of pollen [73]. The different effects of diets on the growth and development of caged *A. cerana* workers should be mainly attributed to the different nutritional composition. It was suggested that the protein supplements were less effective than natural forage because their protein sources are not part of the bees’ natural diet and are therefore less digestible [74]. To explore the mechanisms of different influence, further studies should include more indicators, such as diet digestibility and *vitellogenin* gene expression level to verify the effect of the diets [42].

Honey bees are polylectic and use a variety of pollen sources in their diets. Previous studies have suggested that honey bees normally select a mixed pollen diet, which could reduce the probabilities of protein, vitamin, or mineral deficiencies [75]. Compared to monofloral pollen supply, polyfloral pollen induced higher glucose oxidase activity in newly emerged bees [76] and bees fed on polyfloral pollen lived longer when parasitized by *Nosema ceranae* [27]. Although honey bees are highly dependent on the quality and diversity of floral resources, feeding monofloral pollen is convenient and very common in Chinese beekeeping practice at present. Based on the fact that a diet consisting of multi-flower pollen is preferable [77], research into beneficial diets consisting of polyfloral pollen still needs to be continued.

Factors like climate, geography and pollen availability impact the efficacy of the diets. The results obtained under controlled laboratory conditions may not necessarily reflect the real situation. Both field and cage experiments are necessary to validate the effects of diets on honey bees [63]. Therefore, further experiments are needed to understand the impacts of these different diets on honey bee colony heath, such as palatability, colony productivity, physiological response, and pest and disease response.

Currently, detailed survey data of the amount of pollen substitutes used in Chinese apiculture are lacking. In 2016, Liu et al. [23] stated that only 38% of apiaries in Henan Province feed pollen to their colonies, while 15% of apiaries feed only protein substitutes and 46% feed both pollen and substitutes. In other provinces, an average of 80% of apiaries feed only pollen. However, the pollen pellets collected by honey bees come with several drawbacks, such as expensive cost, the potential that they contain pollutants [78], and disease transmission risks [47]. Therefore, it is becoming more and more common to feed colonies with pollen substitutes. Based on the large number of colonies managed in China, an effective pollen substitute would be of substantial value to the Chinese honey bee industry.

Pollen substitutes have been developed to address pollen deficiencies in colonies during periods of nutrient scarcity. Despite the significant research effort, pollen substitutes do not truly replace natural pollen due to the lack of many nutrients provided by natural pollen resources [21,79]. In the practice of beekeeping in China, beekeepers have used bean pulp, soy flour, yeast powder, or corn protein powder as pollen substitutes. However, the effect of pollen substitutes prepared by these proteins is not ideal. Several studies have been conducted to determine the optimal levels of calcium and phosphorus [80] and the appropriate proportion of wheat germ required in pollen substitutes [81] and to compare the effect of different protein sources, including corn protein powder, rapeseed cake, brewer’s yeast and Candida utilis [82]. Still, a systematic study of pollen substitutes is lacking in China. In addition, research on the effects of diets on honey bee growth and development is mainly focused on *A. mellifera*, and there are few studies on *A. cerana*. In the absence of scientific guidance, beekeepers choose pollen substitutes relying on their personal experience [82]. Therefore, it is urgent to carry out further investigations on the formulation of effective pollen substitutes.

## 5. Conclusions

As the primary source of dietary proteins and lipids, pollen needs to be provided in adequate quantity and quality to maintain healthy honey bee colonies. Nowadays, honey bees are confronted with many stressors, leading to pollen deficiencies, which prompt the use of pollen supplements or pollen substitutes to support colonies. The results in this study suggested that natural pollen resources are more beneficial to *A. cerana* caged workers than the most widely used pollen substitutes in China. In order to draw accurate conclusions, it is necessary to conduct more thorough studies, especially field studies, as this study using caged workers does not account for colony-level interactions that might affect worker metabolism. In addition, studies of diets based on multi-floral pollen are also desirable.

## Figures and Tables

**Table 1:** Information about the four types of pollen pellets used in this study.

Pollen	Producing Region	Longitude (E)	Latitude (N)	Time of Collection	Protein Content (g/100 g)
oilseed rape pollen	Huzhu County, Haidong City, Qinghai Province	101.96°	36.84°	July	24.53 ± 0.40
camellia pollen	Yuncheng District, Ya’an City, Sichuan Province	103.04°	30.01°	March	21.50 ± 0.36
lotus pollen	Dingbian County, Yulin City, Shaanxi Province	107.60°	37.59°	August	18.03 ± 0.42
buckwheat pollen	Guangchang County, Fuzhou City, Jiangxi Province	116.34°	26.84°	October	13.43 ± 0.31

**Table 2:** The pollen substitutes used in this study.

Pollen Substitutes	Ingredients	Protein Content (g/100 g)
Diet 1	vegetable germ, vegetable proteins, yeast powder, lysine, methionine, etc.	23.45 ± 0.01
Diet 2	vegetable proteins, carrot powder, yeast powder, lactic acid bacteria, amino acid, mineral, trace element, multivitamins, etc.	38.16 ± 0.02

**Table 3:** The total diet consumption and the number of workers consuming each diet.

Diet	Total Diet Consumed (g)	Number of Workers
oilseed rape pollen	0.257 ± 0.096 ^ab^	3.3 ± 1.5 ^c^
camellia pollen	0.228 ± 0.034 ^ab^	2.8 ± 0.3 ^c^
louts pollen	0.117 ± 0.008 ^b^	0.5 ± 0.9 ^c^
buckwheat pollen	0.357 ± 0.092 ^a^	13.0 ± 3.4 ^a^
Diet 1	0.135 ± 0.002 ^b^	7.8 ± 1.8 ^b^
Diet 2	0.210 ± 0.018 ^ab^	0.8 ± 0.2 ^c^

The data are mean ± SE. Means in the same column with different letters are significantly different at *p* < 0.05.

## Data Availability

The data presented in this study are available on request from the corresponding author. The data are not publicly available due to privacy concerns and potential political sensitivities.
